# Performance of a new molecular assay for the detection of gastrointestinal pathogens

**DOI:** 10.1099/acmi.0.000160

**Published:** 2020-08-19

**Authors:** Bruce A. Gingras, Jack A. Maggiore

**Affiliations:** ^1^​ IIT Research Institute, Chicago IL 60616, USA; ^2^​ Loyola University Medical Center, Maywood IL 60153, USA

**Keywords:** gastrointestinal testing, gastrointestinal pathogens, molecular detection, stool diagnostics

## Abstract

**Introduction:**

Conventional diagnostic laboratory algorithms for determining the cause of infectious gastroenteritis include culture, biochemical identification and immunoassays. In addition, multiplex PCR-based testing has advanced into the gastroenterology diagnostic arena in recent years.

**Aim:**

The purpose of this study was to evaluate the performance of a new molecular test (Diagnostics Solutions Laboratory GI-MAP) for the detection of bacterial and parasitic pathogens in stool samples spiked with known organisms.

**Methodology:**

Faeces from a healthy human subject were pooled into a standard matrix and screened for the absence of bacteria, parasites and *Helicobacter pylori* antigen. Once confirmed negative single faecal aliquots from the matrix were spiked with solely one pathogen-type from a panel of 14 bacterial pathogens or one of 2 parasitic pathogens at a density of 5×10^6^ organisms ml^−1^. Sixteen spiked samples in appropriate transport media were sent to two testing labs, specifically a reference site using the PCR-based BioFire FilmArray Gastrointestinal Panel, and a second lab using the GI-MAP assay. Seven negative control samples comprised solely of stool matrix were also submitted.

**Results:**

Significant variability was found when the GI-MAP assay was used to test normal stool matrix with and without known bacteria and parasites at densities well within the expected limits of detection. The GI-MAP assay displayed a sensitivity of 80 % and a specificity of only 26 % due to many false positive results. This assay also reported quantitative numbers for pathogens. The BioFire FilmArray Gastrointestinal Panel achieved a sensitivity and specificity of 100 %.

**Conclusion:**

The highly variable results for the GI-MAP assay were unexpected due to the precise pre-spike analysis and the overall maturation of nucleic acid amplification methods within the industry. Problematic to this assay is the poor level of specificity displayed by this assay reporting the presence of several pathogens, which could cause clinicians to treat with antibacterial and/or antiparasitic agents in the absence of any true pathogens.

## Introduction

According to the World Health Organization, there are 1.7 billion cases of gastroenteritis every year and approximately 1.5 million children deaths [1, 2]. The causes of infectious gastroenteritis are viruses (~70 %), bacteria (10–20 %) and parasites (<10 %) [3, 4]. Historically, the methods used to identify these groups of organisms were relatively slow, labour intensive and expensive, making their diagnostic utility confirmatory, at best. Molecular detection of enteric pathogens can provide a comprehensive and rapid alternative to conventional testing. A number of FDA-cleared multiplexed assays are available for use in clinical laboratories and target a wide range of bacterial, viral, and parasitic pathogens, including: BioFire FilmArray Gastrointestinal Panel (BioFire, Salt Lake City, UT, USA) [5], the xTAG Gastrointestinal Pathogen Panel (Luminex Corporation, Austin, TX, USA) [6], the Luminex Verigene Enteric Pathogen Test [7], and the BD MAX Enteric Bacterial, Viral and Parasite Panels (BD Life Science, Parks, MD, USA) [8–12].

The purpose of this study was to determine the performance of a new test (GI-MAP) for the detection of known bacterial and parasitic pathogens in spiked stool samples.

## Methods

An employee of the contributing author’s previous employer (see Author statement) consented to providing a stool matrix sample that was used to construct the experimental specimens for this evaluation. The donor signed an informed consent, which remains on file with the funding agency. The stool matrix from this uninfected healthy subject was screened by an independent reference laboratory using the BioFire FilmArray Gastrointestinal Panel and the *Helicobacter pylori* antigen detection test by Meridian Bioscience (Newtown, OH, USA). All stool samples were negative for the 16 targets (14 bacterial and 2 parasitic) and the *H. pylori* antigen. To minimize the effects of pre-analytical variability, individual stool samples were subsequently homogenized and pooled to create a standard matrix, stored continually at 2–8 °C. Characterized control organisms representing the pathogenic panel in the GI-MA test were used to spike the sample matrix, following aseptic protocols to avoid cross-contamination.

Bacteria were received freeze-dried from the American Type Culture Collection (ATCC, Manassas, VA, USA) (see Table 1) and were grown on Trypticase Soy II blood agar (Becton Dickinson, Cockeysville, MD, USA) and CVA (cefoperazone, vancomycin and amphotericin B) plates (Becton Dickinson, Cockeysville, MD, USA) for *Campylobacter *. Bacterial suspensions were prepared with 0.45 % (w/v) sodium chloride (CareFusion Corporation, San Diego, CA, USA) to a density of approximately 1×10^8^ c.f.u. ml^−1^ using the DensiCHEK Plus instrument (bioMérieux, Hazelwood, MO, USA).

**Table 1.** Results of GI-MAP testing

Organism added to matrix	GI-MAP results*	Spike detection
*** Clostridium difficile* Toxin A+, B+ATCC 9689**	*C. difficile,* Toxin A – High, 1.4×10^3^ c.f.u. gm^−1^ *C*. *difficile,* Toxin B – High, 2.15×10^7^ c.f.u.gm^−1^ *H*. *pylori*† *–* High, 2.5×10^5^ c.f.u.gm^−1^	TP - FP
*** Clostridium difficile* Toxin A-, B- ATCC 700057**	*C. difficile,* Toxin A – less than detection limit (normal) *C. difficile,* Toxin B – less than detection limit (normal)	TN -
*** Clostridium difficile* Toxin A-, B+ATCC 43598**	*C. difficile,* Toxin A – High, 1.9×10^5^ c.f.u. gm^−1^ *C. difficile,* Toxin B – High, 6.32×10^7^ c.f.u. gm^−1^ Enterohemorrhagic *E. coli –* High, 1.13×10^3^ c.f.u. gm^−1^ *H*. *pylori–* High, 2.8×10^5^ c.f.u. gm^−1^ *Vibrio cholerae –* quantity detected but below the normal limit (normal), 5.33×10^0^ c.f.u.gm^−1^	TP TP FP FP Equivocal
*** Escherichia coli* O157:H7 ATCC 35150**	Enterohemorrhagic *E. coli –* High, 5.19×10^5^ c.f.u. gm^−1^ *E. coli O157 –* High, 3.4×10^5^ c.f.u.gm^−1^ Enterotoxigenic *E. coli* LT/ST *–* High, 1.35×10^7^ c.f.u. gm^−1^ *Shiga -like Toxin E. coli* stx1 *–* High, 1.56×10^5^ c.f.u. gm^−1^ *Shiga -like Toxin E. coli* stx2 *–* High, 5.18×10^4^ c.f.u.gm^−1^ *H*. *pylori –* High, 1.7×10^3^ c.f.u.gm^−1^	TP - - - - FP
*** Shigella boydii* ATCC 9207**	*Enteroinvasive E. coli/Shigella –* High, 1.14×10^7^ c.f.u.gm^−1^	TP
*** Escherichia coli* O26:H11 ATCC BAA-2196**	Enterohemorrhagic *E. coli –* High, 1.97×10^7^ c.f.u.gm^−1^ Shiga -like Toxin *E. coli* stx1 – High, 8.84×10^6^ c.f.u.gm^−1^ Shiga -like Toxin *E. coli* stx2 *–* High, 4.99×10^5^ c.f.u.gm^−1^ *H*. *pylori –* less than detection limits (normal)	- TP - Equivocal
*** Escherichia coli* STX1+, STX2- O103:H11 CDC-3008**	Shiga -like *Toxin E. coli* stx1 *–* quantity detected but below the normal limit (normal), 1.56×10^1^ c.f.u.gm^−1^	FN
*** Shigella sonnei* ATCC 29930**	Enteroinvasive *E. coli/Shigella –* High, 4.22×10^6^ c.f.u. gm^−1^	TP
*** Salmonella bongori* ATCC 43975**	*Salmonella –* High, 3.48×10^8^ c.f.u.gm^−1^	TP
*** Vibrio cholerae *ATCC 25870**	*Vibrio cholerae –* quantity detected but below the normal limit (normal), 1.24×10^3^ c.f.u.gm^−1^ Enterohemorrhagic *E. coli –* quantity detected but below the normal limit (normal), 2.4×10^2^ c.f.u.gm^−1^ *H*. *pylori –* High, 1.3×10^4^ c.f.u. gm^−1^	FN Equivocal FP
***Salmonella Enteritidis*ATCC 13076**	*Salmonella –* High, 2.89×10^7^ c.f.u. gm^−1^ Adenovirus *–* quantity detected but below the normal limit (normal), 9.15×10^7^ c.f.u. gm^−1^ *H. pylori –* High, 9.6×10^3^ c.f.u. gm^−1^	TP Equivocal FP
*** Yersinia enterocolitica* O:8 ATCC 9610**	Enterohemorrhagic *E. coli* – quantity detected but below the normal limit (normal), 4.99×10^1^ c.f.u. gm^−1^ *Yersinia enterocolytica -* quantity detected but below the normal limit (normal), 2.58×10^2^ c.f.u. gm^−1^ *Giardia –* High, 3.64×10^4^ c.f.u.gm ^−1^ *H*. *pylori* – High, 3.5×10^6^ c.f.u. gm^−1^	Equivocal FN FP FP
*** Campylobacter jejuni* ATCC 33560**	*Campylobacter –* High, 2.96×10^6^ c.f.u.gm^−1^	TP
*** Campylobacter coli* ATCC 51729**	*Campylobacter –* High, 1.33×10^6^ c.f.u.gm^−1^	TP
***Giardia intestinalis***	*Giardia –* High, 1.43×10^6^ c.f.u. gm^−1^	TP
***Cryptosporidium parvum***	*Cryptosporidium –* High, 5.48×10^5^ c.f.u. gm^−1^	TP
**Negative control**	Negative	TN
**Negative control**	Negative	TN
**Negative control**	*Giardia -* quantity detected but below the normal limit (normal), 4.10×10^3^ c.f.u. gm^−1^	TN
**Negative control**	Enteroinvasive *E. coli/Shigella –* High, 1.53×10^2^ c.f.u. gm^−1^ *H*. *pylori* – High, 1.13×10^3^ c.f.u. gm^−1^	FP FP
**Negative control**	* H. pylori* – High, 2.0×10^3^ c.f.u. gm^−1^	FP
**Negative control**	* H. pylori* – High, 9.0×10^3^ c.f.u. gm^−1^	FP
**Negative control**	Enterotoxigenic *E. coli* LT/ST – High, 6.92×10^8^ c.f.u. gm,^−1^	FP

*GI-MAP Laboratory report lists pathogens results as:<dl, (normal); a quantity detected with Log_10_ quantitation but below ‘normal’ quantity; or a quantity detected with Log_10_ quantitation note as ‘high’.

†Independent reference laboratory using the Meridian *H. pylori* antigen detection test.

TP, true positive; TN, true negative; FP, false positive; FN, false negative.

*Cryptosporidium parvum* and *Giardia duodenalis* (*lamblia/intestinalis*) were received in phosphate buffered saline (PBS) with antibiotics and 0.01 % (v/v) Tween 20 (Waterborne, New Orleans, LA, USA). *C. parvum* was supplied at approximately 5×10^8^ oocysts ml^−1^ and *G. duodenalis* at approximately 1×10^8^ cysts ml^−1^.

MCC Para-Fix C and S Medium (Modified Cary Blair) transport media (Medical Chemical Corporation, Torrence, CA) and Para-Pak C and S vials (Meridian Bioscience, Cincinnati, OH, USA) were inoculated in pairs with 5 ml of the homogenized stool matrix then mixed well. These vials were used as negative controls for this study.

Then, 1 ml of Cary Blair transport media was aseptically removed from each vial that was to be spiked in pairs with the bacteria or parasite pathogen. Subsequently 1 ml of the suspension of each of the pathogens listed in Table 1 was aseptically added to the vial, followed directly with 5 ml of the stool matrix for a final pathogen density of approximately 5×10^6^ organism ml^−1^. This level was chosen to exceed normal limits of detection seen in multiplexed PCR assays (approximately 3×10^4^ organisms ml^−1^). The volume of homogenized stool added to the negative controls and spiked specimens was the same for all vials.

Twenty three samples were shipped at room temperature by overnight courier to a reference laboratory and were tested within 4 days of preparation using the PCR-based BioFire FilmArray Gastrointestinal Panel to independently confirm expected results. The BioFire was utilized as a qualitative comparator assay, but not intended to serve as a true ‘gold standard’ comparison, even though it has established detection limits and published sensitivity/specificity data from other clinical trials [6, 7]. Paired vials were placed in individual test kits and shipped the same day at room temperature by overnight courier to Diagnostic Solutions Laboratory (Alpharetta, GA, USA) for testing using the GI-MAP assay. This assay, as indicated by this company, was developed and the performance characteristics determined by Diagnostic Solutions Laboratory.

GI-MAP Laboratory reports pathogen results as: ‘<dl’, a quantity detected with Log_10_ quantitation but below ‘normal’ quantity. A quantity detected with Log_10_ quantitation above the ‘dl’ is noted as ‘high’. For comparison to inoculated samples, and reference laboratory results, GI-MAP results of <dl and results with a quantity detected with Log_10_ quantitation but below ‘normal’ quantity were considered *negative* and results a quantity detected with Log_10_ quantitation note as ‘high’ were considered *positive*. At the time of this study, GI-MAP testing reports the detection of 12 bacterial pathogens, three parasitic pathogens and three viral pathogens. The company indicates that the results are reported as c.f.u. per gram of stool as determined by PCR. (https://www.diagnosticsolutionslab.com/tests/gi-map; accessed 2 July 2020).

## Results

The results from the GI-MAP test are presented in Table 1. GI-MAP detected the organisms added to the matrix in 12 of 16 spiked samples, with a sensitivity of 80 %. Eleven of 23 total samples were reported as ‘high’, with Log_10_ quantitation above the normal limits for organisms that were not supplanted to the sample and were not detected by the BioFire assay. Because of multiple organisms being detected by this assay in the 23 samples, an overall specificity of just 27 % was realized. Of the seven samples with no organisms added, two were correctly reported as nothing detected (<dl), four were reported as a pathogen detected with high levels (one sample with two pathogens detected), and one sample as quantity detected but the quantity was below normal limits. All samples spiked with *C. difficile* were accurately detected by GI-MAP, as were the samples spiked with *Salmonella bongori *, *Shigella sonnei *, *Campylobacter jejuni, Campylobacter coli, Giardia intestinalis* and *Cryptosporidium parvum*. However, samples spiked with *Yersinia enterocolitica* and *Vibrio cholerae* were not detected by GI-MAP, and false positivity was encountered in several aliquots for *H. pylori,* and in individual negative controls sample aliquots for Enterotoxigenic *E. coli* and Enteroinvasive *E. coli *.

The BioFire FilmArray rendered a sensitivity of 100%, no false negatives, and a specificity of 100 %, no false positives (data not shown). Contrastingly, only one virus was reported by GI-MAP in one sample, Adenovirus, at a quantity of 9.15×10^7^ c.f.u. gm^−1^ (Table 1). The sample matrix was negative for Adenovirus using the BioFire assay.

## Discussion

There is a growing demand for faster results for microbiology testing as well as an increasing interest and application of molecular based assays. Studies have reported the successful application of molecular methods for detection of micro-organisms from human gastrointestinal samples [8–14]. To our knowledge, no previous studies have been conducted to evaluate this test. In this study we examined the ability of a novel DNA method for identifying pathogenic micro-organisms in human stool samples.

**Fig. 1.**
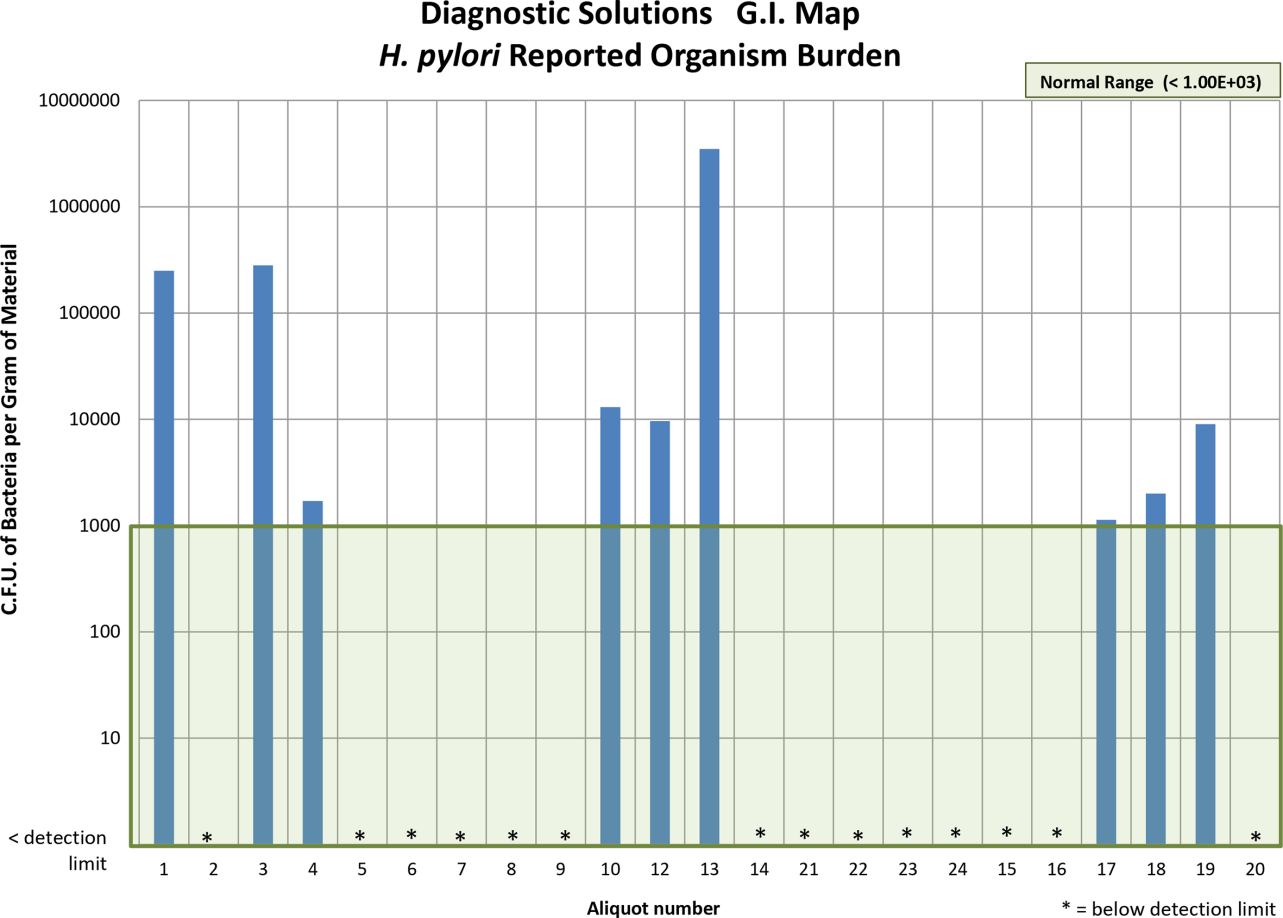
Sample burden for *H. pylori *.

The highly variable results from this study were unexpected given the protocols employed to minimize pre-analytical variability and maturity of the field of nucleic acid amplification for the detection of stool pathogens. Limits of detection and primer differences are well known to affect PCR testing results but these factors do not explain the number of false-positive results in the absence of added target. The report of *H. pylori* by GI-MAP in nine samples as ‘high’ by PCR could not be confirmed due to the lack of a reference PCR assay, however, the use of the *H. pylori* antigen detection test was consistently negative among these same 20 aliquots. See Fig. 1, which illustrates this unanticipated degree of *H. pylori* variability among 23 aliquots expected to show a negative outcome for this pathogen.

Quantitative multiplex PCR in stool, like that in the GI-MAP DNA Stool Analysis assay, has no established clinical relevance. Quantitation of target in stool as shown in Table 1, and Fig. 1 is extremely variable, as the volume and density of stool is affected by many factors, including diet, liquid consumption, and other medical conditions. This assay reports organism densities in c.f.u. gm^−1^of stool. However, standardizing this type of assay to the dry weight of stool is technically difficult and would still result in tremendous variability with diet, hydration and fibre intake. In addition, there are a significant number of variables associated with stool such as PCR inhibitors, pH, protein concentrations, primer affinity and other highly variable factors that can affect the reaction. Semi-quantitative PCR assays have recently been developed and tested using specimens where the volume can be controlled such as with bronchoalveolar lavage samples, namely the BioFire FilmArray Pneumonia Panel.

Importantly, the pathogenesis of most enteric pathogens is not dependent on quantity, and any detectable amount of pathogen is indicative of clinical infection. Detection below ‘threshold of normal’ is a result that may be misleading and likely represents assay background. This reporting should not be interpreted as low-level presence of pathogens, as there is no established low-level of pathogenic organisms such as *Vibrio, Salmonella* or *Shigella* that is acceptable or not associated with disease. In this study for example, in the sample to which *Vibrio cholerae* ATCC 25 870 was added to matrix and detected by BioFire, *Vibrio cholerae* was reported by GI-MAP as detected, but with a level lower than normal, giving a false-negative reading with a concentration of 1.24×10^3^ c.f.u. gm^−1^ of stool. This is below the provided lower limit of normal: <1.00×10^5^ c.f.u. gm^−1^. The presence of any amount of *V. cholerae* in a patient with symptoms is generally thought to be the cause of the disease and warrants treatment. In the sample with *Clostridium difficile* Toxin A-, B+ATCC 43598, GI-MAP reported the sample as having *V. cholerae* detected at a level of 5 c.f.u. ^−1^, which is a detection level that is difficult to scientifically justify.

There were limitations in the present study. The initial stool sample/matrix was only tested by the BioFire FilmArray GI panel and the *H. pylori* antigen detection test by Meridian Bioscience. Both screening assays have specific limits of detection, sensitivity and specificity that may be different from the test assay and therefore testing results may not match. Specifically, there is currently no FDA-approved PCR method for the detection *H. pylori *, therefore it was decided to use a commonly accepted, FDA-approved method as a comparator. In addition, the design of this study allowed a single consistent matrix and a limited number of replicates. The lack of comparator quantitative PCR assays prevented a direct comparison with the quantitative results of the GI-MAP. Additional data would be needed to calculate positive and negative percent agreement with a gold-standard reference method to validate the false-positive and false-negative finding in the present study. In this study’s qualitative analysis the GI-MAP assay could not attain the sensitivity of the BioFire multiplex assay, and its specificity was surprisingly low. The extreme number of false positives could lead clinicians to treat patients in the absence of any true pathogen.

Although there is a need to develop rapid molecular testing assays for characterization of the gut microbiome, physicians and patients need to be aware that all stool analysis assays may not provide consistent results with both false-positive and false-negative results possible. The clinical implications for diagnosis and treatment of gastroenteritis is potentially significant because of missed diagnoses, and the use of antibacterial or antiparasitic agents in the absence of true infections. The limitations of the GI-MAP method reported here may restrict its applications for the diagnosis of gastroenteritis.
